# Strategies to prevent intraoperative lung injury during cardiopulmonary bypass

**DOI:** 10.1186/1749-8090-5-1

**Published:** 2010-01-11

**Authors:** Efstratios E Apostolakis, Efstratios N Koletsis, Nikolaos G Baikoussis, Stavros N Siminelakis, Georgios S Papadopoulos

**Affiliations:** 1 Department of Cardiothoracic Surgery, University of Patras, School of Medicine, Patras, Greece; 2 Department of Cardiac Surgery, University of Ioannina, School of Medicine, Ioannina, Greece; 3 Department of Clinical Anaesthesiology and Intensive Postoperative Care Unit, University of Ioannina, School of Medicine, Ioannina, Greece

## Abstract

During open heart surgery the influence of a series of factors such as cardiopulmonary bypass (CPB), hypothermia, operation and anaesthesia, as well as medication and transfusion can cause a diffuse trauma in the lungs. This injury leads mostly to a postoperative interstitial pulmonary oedema and abnormal gas exchange. Substantial improvements in all of the above mentioned factors may lead to a better lung function postoperatively. By avoiding CPB, reducing its time, or by minimizing the extracorporeal surface area with the use of miniaturized circuits of CPB, beneficial effects on lung function are reported. In addition, replacement of circuit surface with biocompatible surfaces like heparin-coated, and material-independent sources of blood activation, a better postoperative lung function is observed. Meticulous myocardial protection by using hypothermia and cardioplegia methods during ischemia and reperfusion remain one of the cornerstones of postoperative lung function. The partial restoration of pulmonary artery perfusion during CPB possibly contributes to prevent pulmonary ischemia and lung dysfunction. Using medication such as corticosteroids and aprotinin, which protect the lungs during CPB, and leukocyte depletion filters for operations expected to exceed 90 minutes in CPB-time appear to be protective against the toxic impact of CPB in the lungs. The newer methods of ultrafiltration used to scavenge pro-inflammatory factors seem to be protective for the lung function. In a similar way, reducing the use of cardiotomy suction device, as well as the contact-time between free blood and pericardium, it is expected that the postoperative lung function will be improved.

## Introduction

Despite the improvement in the cardiopulmonary bypass (CPB) techniques as well as the postoperative intensive care, impaired pulmonary function is a well-documented (by enormous experimental and clinical evidence) complication of cardiopulmonary bypass, resulting in increased morbidity and mortality [1-3]. However, whether CPB itself is directly responsible for the whole postoperative lung dysfunction is still controversial. It is indirectly suggested by some studies following off-pump coronary artery bypass, which although an attenuated inflammatory response has been shown, the degree of postoperative lung dysfunction was similar with that of conventional Coronary Artery Bypass Grafting CABG [4,5]. Namely, for this postoperative pulmonary dysfunction CPB may not be the only factor contributing, but other factors related to the cardiac operation such as anaesthesia, temporary cardiac dysfunction, infused catecholamines, altered mechanical of thoracic cage, etc could play an important role [3,6-11]. The reported increased mortality and morbidity of this early postoperative pulmonary dysfunction after cardiac surgery may be related to the duration of mechanical ventilation, neurological, renal and infectious complications, ICU and hospital stays, and subsequently increased mortality [12]. Despite the well-documented impairment of pulmonary function even after uncomplicated CPB, effective precautions and ideal management strategies for this problem are still under debate [3,4]. The scope of this review is, therefore, to highlight the path of genetic and pathophysiological mechanisms involved in this injury, and the possible perioperative therapeutic options and manipulations that could be implemented, in order to alleviate the expected postoperative lung dysfunction.

## Methodology and strategy for management of lung dysfunction after cardiac surgery

### 1. Prevention and management of the inflammatory reaction due to CPB

Since the inflammatory response of CPB is multifactorial, a combined therapeutic approach should be implemented for the attenuation of the clinical sequelae. On the one hand, the abrogation of CPB by using Off-Pump techniques alone is not possible in many cases, and on the other hand, this technique alone does not seem to fully alleviate postoperative lung dysfunction [13,14]. Other modifications of CPB techniques, such as the utilization of heparin-coated circuits, use of ultra-filtration techniques or the use of the Drew-Anderson technique, may be beneficial for a reduction in the observed activation of systemic inflammatory response syndrome (SIRS) or the scavenging of various pro-inflammatory cytokines [4,15,16].

#### 1.1 Inversion to Off-Pump operations

Although CPB causes disturbances in lung mechanics, it may not be on its own a major contributor to the observed postoperative gas exchange abnormalities following heart operations [3,17,18]. To date the experimental and clinical data comparing On-pump and Off-pump surgery suggest an affected cardiac function in favour of Off-Pump operations, expressed by a reduced tissue oxygenation, a phenomenon which might be related to a greater myocardial damage during hypothermic CPB operations [14,19-21]. In addition, the higher lactate levels in the CPB group suggest greater tissue O_2 _demands after hypothermic CPB perfusion in comparison with those demands with Off-pump surgery [22]. Although initial studies showed reduction in indexes of systemic inflammation after OPCAB and pulmonary complications [23], the negative influence of CPB on the lungs, is not apparent by comparing conventional CABG with Off-Pump Coronary Artery Bypass (OPCAB). Indeed, some clinical studies showed that, both On-pump and Off-pump CABG patients experienced similar degrees of decreased PaO_2 _and increased P(A-a)O_2_, but a higher percentage of pulmonary shunt fraction after On-pump operations [17,18,24]. However, a randomized study by Staton et al [25] compared the postoperative lung function after OPCAB and conventional CABG, concerning fluid balance, hemodynamics, arterial blood gases, chest radiographs, spirometry, pulmonary complications, and extubation-time. Paradoxically, postoperative compliance was reduced more after OPCAB, and fluid balance was significantly higher in the same group. Despite these changes, immediate postoperative PaO_2 _on FiO_2 _of 1.0 was significantly higher after OPCAB and extubation-time was significantly shorter, while the postop-chest radiographs, spirometry, mortality, re-intubation, or re-admission for pulmonary complications, were not significantly different between groups [25]. In conclusion, although it is impossible to perform all the heart operations without CPB, this hypothetical inversion alone cannot prevent systemic inflammatory reaction and lung function impairment. Although this scenario can abolish the negative effects of CPB on lung function it is not able to diminish completely the pro-inflammatory factors that are produced, despite the fact that the postoperative lung impairment seems to be generated to a lesser extent.

#### 1.2 Heparin-coated circuits and new-technology circuits

The hostile surface of extracorporeal circuit is considered to be a major factor of inflammatory reaction. Over the last years a large improvement has been observed in the construction and the clinical use of circuits lined with more biocompatible coating. The following have been used as coating materials: heparin [4,15,16], poly-2-methoxyethyl acrylate [26], synthetic protein [27], and phosphorylcholine [28]. The first and most extensively studied coating material used is that of heparin. The concept behind heparin coating is to mimic the endothelial surface that contains heparin sulphate [2]. Hence, the main beneficial effects of heparin-coated circuits are considered to be the following two: first, a reduction of complement activation (and mainly of factor C5a) ranging between 25% and 45% [29,30], and second, a reduction of the inflammatory reaction which is thought to be accomplished in two ways: through a reduction of complement activation, and through binding of phospholipase A_2 _[31]. Heparin reduces the inflammatory responses especially as far as the actions of platelets, leukocytes, and endothelial cells are concerned [31-34]. This effect is noticeable by a decreased production of IL-6, IL-8, E-selectin, lactoferin, myeloperoxidase, integrin, selectin, and platelet β-thromboglobulin release, and reduced production of oxygen free radicals, as well [31-34]. Concisely, all the above described effects of heparin-coated circuits should have beneficial impact on clinical outcomes. Indeed, a clinical study showed a decreased intrapulmonary shunt with improved respiratory index (PO_2_/FiO_2_) after CPB by using heparin-coated circuits, although intubation time and ICU stay were not affected [35]. Others, using a scoring-system based either on intubation time, the central-peripheral temperature difference, the postoperative fluid balance, and on various adverse effects after CABG, showed a significantly positive clinical effect in patients treated with heparin-coated circuits, and especially in patients with cross-clamp times exceeding 60 min [16,36]. De Vroege et al [31] demonstrated comparatively significant postoperative differences in favour of the patients treated with heparin-coated circuits in terms of the pulmonary shunt fraction, the pulmonary vascular resistance index, and the PaO_2_/FiO_2 _ratio, as well as various inflammatory markers reflecting complementary activation. In addition, they found reduced activation of pulmonary capillary endothelial cells in the same group of patients, suggesting that the heparin-coated circuit may have beneficial effects on pulmonary function [31]. Compared with conventional circuits, the heparin-coated may improve lung compliance and pulmonary vascular resistance and thus reduce intrapulmonary-shunt [37]. However, most clinical studies have shown, that these beneficial effects did not influence the intubation-time or the ICU-stay of patients [31,37,38]. Furthermore, in contrast to initial expectations, thrombin generation and the activity of the fibrinolytic system were not reduced using heparin-coated circuits [39]. Recently, Speekenbrink et al [40], proposed a novel miniaturized CPB system with the aim to attenuate lung and other organ dysfunction, and generally to diminish the inflammatory reaction and the derangement of patient homeostasis. The principles of this system described also by others [40-43] are the following: it uses a low prime volume of only 800 versus 2000 ml for the conventional system; all of circuit components are heparin-coated and primed with aprotinin; it is a closed-volume system;, it uses an additional pump for the venous line;, and in addition, it uses a "controlled-suction' system, or a "cell-saving" system, to minimize the contact-time between blood and non-endothelialized tissues. A large amount of the priming volume can be extracted from the extracorporeal circuit by "controlled exsanguinations" of the patient into the circuit, and as a result the unpleasant hemodilution may be reduced [40]. By using his system, the reduction in complementary activation is reduced by 25 to 45% and as a result, the expected impairment on lung function is reduced [40]. Nollert at al [44] compared the outcomes with conventional CPB and miniaturized cardiopulmonary bypass after CABG in 30 patients, concerning the inflammation and coagulation, measuring levels of IL-2, IL-6, IL-10, TNF, CRP, WBC differentiation, d-dimers, fibrinogen, and platelet's number. Surprisingly, they did not find any significant difference of any parameter of inflammation or clinical outcomes (blood loss, need for blood products, ICU-stay and hospital-stay) amongst the two groups. However, in two cases dangerous air leaks occurred in the closed miniaturized circuit, suggestive of a more narrow safety margin. Therefore, the expected protective effect on lung function by using these systems seems to be insufficient for broad clinical use at the time this review is written.

#### 1.3 Leukocyte depletion

Since experimental studies have documented that leukocytes were entrapped into the capillaries of lungs [45] and play an important role in the inflammatory reaction after CPB, their depletion during CPB, may be beneficial. Indeed, experimental studies showed that leukocyte depletion by filtration reduced heart and lung reperfusion injury [45]. However, clinical comparative studies have shown ambiguous results. Some of them showed better preserved lung function and reduced free oxygen radicals production following CPB, expressed by improved PaO_2 _[45-47] while others did not show any difference [48,49] despite the reduced IL-8 production [48]. Other studies have shown, that, although the leukocyte depletion filter of the arterial line removes leukocytes from the circulation, the systemic neutrophil count may [49,50] or may not be reduced [51]. A randomized study compared the effectiveness of leukocyte filter depletion with a common arterial filter, in patients undergoing conventional CABG. They found significantly better oxygenation indices; lower extravascular lung water scores, and less duration of postoperative mechanical ventilation in the leukocyte depletion filter group [52]. In addition, leukocyte filtration did not offer any significant preservation of lung function, for CPB-time less than 90 minutes. Warren et al [53], in their extensive review examined the effectiveness of several leukocyte depletion filters, used in cardiac surgery. They concluded that: a) whilst the filters did not appear to significantly lower leukocyte count, they may preferentially remove activated leukocytes, b) a small improvement in lung function is evident early postoperatively, but this does not lead to decrease mortality or better clinical outcomes, c) their use attenuates the reperfusion injury at the cellular level, but without substantial clinical improvement, and d) up to date there are no evidence-based data to support the routine use in cardiac surgery.

#### 1.4 Ultrafiltration

Ultrafiltration was used in cardiac surgery for removing volume of priming and reducing the postoperative oedema, the total body water, but specifically that of lungs resulting in better oxygenation postoperatively [54,55]. Besides this function, it has been postulated that ultrafiltration may remove also destructive and inflammatory substances from the circulation, inflammatory cytokines, and scavenge toxins [56]. Indeed, various studies have shown that by using ultrafiltration the levels of IL-6, IL-8, as well as systemic oedema formation, or pulmonary hypertension can be effectively reduced, while concomitant improvement of the lung function (reduced alveolar-capillary oxygen pressure gradient) is recorded [56-58]. Another comparative study in children showed, that the conventional ultrafiltration resulted in a significant immediate improvement in static lung compliance and dynamic lung compliance, as well as gas exchange capacity. However, this effect is observed only for the first 6 postoperative hours and did not result in significant improvement of clinical outcomes (intubation-time, ICU-stay, or hospital-stay) [57]. A similar comparative study^54 ^showed that: a) the pulmonary function was improved via a significantly increased pulmonary compliance, a decreased airway resistance and an improved pulmonary gas exchange after CPB, as reflected by a decreased alveolo-arterial oxygen gradient, b) the levels of serum IL-6 in the modified ultrafiltration group were much lower than in the control group, c) the thromboxane B2 was significantly removed by ultrafiltration contributing to a lower lung vessels permeability, and, finally, d) ultrafiltration did not affect the levels and the action of endothelin-1. Finally, the main advantage of ultrafiltration seems to be, in our opinion, the desirable increase of colloid oncotic pressure which subsequently prevents the development of pulmonary interstitial oedema.

#### 1.5 Hemodilution

The mixing of the priming solution with the patient's own blood at the beginning of CPB results in an abrupt hemodilution [48]. This hemodilution is desirable, since it facilitates the tissue-perfusion. However, if the hematocrit is restored below a level of 23%, it has been shown to contribute to an increased interstitial oedema in vital organs (e.g., brain, lungs, myocardium), resulting in increased mortality [59]. Consequently, by increasing the colloid oncotic pressure of the priming solution (replacement of crystalloids with colloids), Jansen et al showed that the postoperative course was improved and the hospital-stay significantly reduced [60]. Another study showed that better hemodynamic parameters such as arterial pressure, cardiac index, and vascular resistance, and higher oxygen delivery can be achieved by the reduction of priming volumes [61].

Similarly, other methods used to prevent excessive hemodilution during extracorporeal circulation, such as the use of blood cardioplegia or perioperative hemofiltration, showed even further reduction of blood transfusions [40].

In conclusion, clinical data suggests that the most important result of "controlled hemodilution" contribute to a reduced interstitial lung oedema and therefore to an improvement of postoperative lung function.

#### 1.6 The cardiotomy suction

Various studies have shown that the collected pericardial blood during the cardiac operations using CPB, is activated by tissue plasminogen activator (t-PA), while it has been additionally found to contains pro-coagulants and platelets factors [40,62]. However, this does not mean that this specific blood is partially activated or that it contains fibrinogen degradation products, and, that its re-transfusion may interact with platelets to form undesirable complexes, and derangements of haemostasis [40]. Indeed, various clinical studies have confirmed that the re-transfusion of blood collected in the pericardium during CPB induces a dose-dependent inflammatory response, impairs hemostasis, enhance various inflammatory reactions, and also impair the postoperative lung function [63,64]. In order to reduce this cascade of activation of pericardial blood, various techniques have been proposed. First, a reduction of time between the contact of shed blood with the pericardium and its re-transfusion might diminish the induced inflammatory reaction [40,65]. Second, the use of a controlled suction device which incorporates a level sensor that is activated only when blood accumulates in the pericardium, minimizes air entering into the suction line, and thus the formation of activating air-blood interfaces [40]. Third, the topical administration of aprotinin into the surgical wound and the pericardium has been shown to inhibit the hyper-fibrinolysis that occurs in the pericardial blood which in turn leads to improved hemostasis [66]. Finally, since heparin levels in the re-aspirated pericardial blood have been shown to be lower than systemic levels, topical administration of heparin might also reduce the activation of pericardial blood, by reducing thrombin activity [67].

#### 1.7 Pharmacological manipulations

##### Corticosteroids

An experimental study showed that after pre-treatment with methylprednisolone the postoperative lung function, expressed by alveolar-arterial oxygen gradient, pulmonary vascular resistance, and extracellular lung water, was improved [68]. In a similar way, clinical studies have shown that administration of corticosteroids before CPB inhibits the production of pro-inflammatory cytokines IL-6, IL-8, and TNFα, while it simultaneously increases the IL-10 levels, which exerts an anti-inflammatory action [16,69]. Other studies showed that methylprednisolone administration can inhibit neutrophil CD11b expression and neutrophil complement-induced chemotaxis, thereby decreasing neutrophil activation and post-CPB neutropenia [4,70-72]. In contrast, other clinical studies did not obtain to confirm the superiority of methyl-prednisolone administration during cardiac surgery concerning the postoperative alveolar-arterial oxygen gradient, the pulmonary shunt, the lung compliance or the intubation-time [73,74]. However, although evidence-based guidelines are still lacking, some authors remain adherents of steroid administration and consider it as a "fundamental strategy" in their fast-track recovery protocol [4,15,72].

##### Aprotinin

Hill et al in a clinical study described that the administration of aprotinin in patients following CPB reduced the levels of TNF-α, neutrophil elastase release, complementary activation, neutrophil CD11 upregulation, as well as lower IL-8 levels in the bronchoalveolar lavage (BAL) fluid and pulmonary neutrophil sequestration [71,75]. Others reported that these effects of aprotinin on the inflammatory response to CPB were dose dependent [76]. Specimens from the lung of patients receiving aprotinin before CPB contained reduced levels of of malondialdehyde, a marker of oxygen free radical damage, higher glutathione peroxidase levels, and reduced leukocyte sequestration [77]. The addition of aprotinin in the priming solution in recipients undergoing heart transplantation showed, that the inflammatory response, and in particular the postoperative pulmonary dysfunction, were both attenuated, resulting in a reduced postoperative morbidity and ICU-stay [78].

##### Heparin

Heparin is nowadays still considered as absolutely necessary for open heart operations. On the other hand, studies have shown that heparin administration a), results in a rapid release of t-PA from its body sources, which may induce fibrinolysis [79], b) causes (in vitro) inhibition of platelet function in more than 30% of patients, thus leading to increased postoperative blood loss [80], c) has pro-activating properties on granulocytes and platelets [81], and finally d), heparin after its neutralization with protamine, is inducing an activation of the complement system, action which is correlated with postoperative pulmonary shunt fraction [82]. To avoid these adverse effects of heparin, some possible alternatives have been proposed. The recombinant form of platelet-factor 4, which binds and subsequently inhibits heparin, could be used as an attractive alternative to protamine [83]. Recombinant hirudin, a selective thrombin inhibitor derived from leeches, is another possible attractive alternative [40], which has shown in experiments good clinical results without increased bleeding tendency [40,84]. However, disadvantages from the use of recombinant hirudin are the absence of specific antidote, the possible activation and depletion of other factors of the coagulation cascade, as well as it does not completely inhibit the formation of thrombin [40]. Therefore, heparin still remains irreplaceable but possibly in the near future there might be a role for hirudin as an adjunct to heparin.

##### Monoclonal anticytokine antibodies

To date some authors believe, that in the near future the perioperative administration of monoclonal *anticytokine *antibodies which reduce the levels of pro-inflammatory cytokines during open heart operations, might attenuate the harmful influence of CPB on the lungs [5,15,40].

#### 1.8 Continuing ventilation during CPB

Apnoea during CPB has been suggested to promote activation of lysosomal enzymes in the pulmonary circulation, which in turn are correlated with the incidence of postoperative pulmonary dysfunction (ALI or ARDS) [85]. To prevent this dysfunction, it has been applied some maneuvers such as the intermittent ventilation or application of continuous airway pressure (CPAP) during CPB [5,40,86]. CPAP application during CPB has been reported as an effective adjunct in some studies [86,87]. However, others reported either no difference, or a non-significant difference lasting less than 4 to 8 hours between patients treated with CPAP compared to controls [9,88,89]. Maintaining ventilation together with pulmonary artery perfusion during CPB has been proposed as another option to attenuate the post-CPB impairment of lung function. Indeed, Friedman et al [90] in an experimental comparative study showed that ventilation with pulmonary artery perfusion during CPB should have a beneficial role in preserving lung function, possibly by reducing platelet and neutrophil sequestration and attenuating the TXB_2 _response after CPB. In contrast to this, another experimental study showed that continuous ventilation during CPB provided no significant improvement in pulmonary vascular resistance, respiratory index, or oxygen tensions [91]. More recently, John et al [92] showed in their randomized study that continued ventilation during CPB by tidal volume of 5 ml/Kg resulted significant smaller extravascular lung water and a shorter extubation-time. To date, the evidence for clear benefits of maintaining ventilation alone during CPB is inconsistent, with most studies showing no significant preservation of lung function [5,88]. Similarly, no differences in pulmonary membrane permeability were found between ventilated and non-ventilated patients undergoing CPB [93].

### 2. Prevention and management of other (except of cardiopulmonary bypass) causes of lung dysfunction

Indirect factors of lung dysfunction are the ischemia and reperfusion of the heart, which have been linked with increased production some pro-inflammatory factors [29,94,95]. Myocardial cooling and cardioplegia perfusion have been shown to attenuate the negative effects of ischemia on the heart after cross-clamping of the aorta, by reducing the metabolic demand of the myocardium [40]. Nevertheless, ischemia will occur or is already present owing to the disease process that is being treated. The ischemia will consume high-energy phosphate of cells and may cause a degree of reversible or irreversible myocardial damage [40]. Proposed mediators of reperfusion injury following ischemia involve the generation of oxygen free radicals produced via the xanthine oxidase reaction. Exposure of the ischemic endothelium to these radicals induces a rapid up-regulation of P-selectin and integrin expression [96]. At the beginning of reperfusion this will result in the accumulation of more activated neutrophils, which shed their cytotoxic enzymes, cytokines, and oxygen free radicals on the endothelium, leading finally to an extensive tissue injury [40]. Damage to receptors involved in the activation of nitric oxide (NO) synthase will reduce NO production which may produce coronary spasm and the no-reflow phenomenon [97,98]. Possible ways to reduce reperfusion injury include maintenance of physiological oxygen concentration during CPB, oxygen radical scavengers administration, inhibition of xanthine oxidase by allopurinol, as well as drastic reduction of ischemia by using continuous warm blood cardioplegia techniques [99-102].

## Conclusions

It is clear that many factors are involved in the detrimental effects of CPB in all organs and especially in the lungs [3]. Therefore, substantial improvements in the process of CPB can only be obtained when a multi-factorial approach is followed, directed at both material-dependent and material-independent factors [40]. There is a huge research to this direction and most of the results are still debatable. However, we could herein summarize the most important beneficial manipulations.

a) By abolition of CPB or by reducing as much as possible its time, a better postoperative lung function is expected [103,104].

b) By minimizing the extracorporeal-circuit surface area (miniaturized-circuits), the endothelial injury, the granulocytes sequestration and its activation is expected to be much lower [105,106].

c) By replacement of circuit-surfaces with "biocompatible" surfaces as these of heparin-coated, and material-independent sources of blood activation, the expected post-CPB lung injury should be lower [31,40].

d) By maintaining pulmonary artery perfusion during CPB, the lung ischemia is prevented [15,90,107,108].

e) By using "lung-protective" medication such as corticosteroids and aprotinin, the lungs should be protected against the toxic influence of CPB [4,72,77,102].

f) By using selectively the Drew-Anderson technique to abrogate the xenograft oxygenator, the reduced granulocyte sequestration in the lungs and the minimal complement activation preserve a better postoperative lung function [109,110] the font was corrected here

g) By using (conventional or modified) ultrafiltration during CPB, some pro-inflammatory factors especially "toxic" for the lung function are scavenged [54-56].

h) By drastic reduction of cardiotomy suction to the minimum or by using a controlled cardiotomy suction system which minimizes superfluous suctioning and air entering the pericardial suction line, the postoperative lung function is significantly preserved [48,62-65].

i) By using leukocyte depletion filters for expected long-lasting CPB-time (>90 minutes), a reduced free oxygen radicals production and a better preserved lung function can be achieved [5,52,53].

j) By meticulous application of rules of myocardial protection (during ischemia and reperfusion) the lungs are indirectly protected from several pro-inflammatory factors produced during this process [96,101].

## Competing interests

The authors declare that they have no competing interests.

## Authors' contributions

All authors: 1. have made substantial contributions to conception and design, or acquisition of data, or analysis and interpretation of data; 2. have been involved in drafting the manuscript or revisiting it critically for important intellectual content; 3. have given final approval of the version to be published.
